# Structural basis for mTORC1 regulation by the CASTOR1-GATOR2 complex

**DOI:** 10.21203/rs.3.rs-5073364/v1

**Published:** 2025-05-13

**Authors:** Rachel M. Jansen, Clément Maghe, Karla Tapia, Selina Wu, Serim Yang, Xuefeng Ren, Roberto Zoncu, James H. Hurley

**Affiliations:** 1 Department of Molecular and Cell Biology, University of California Berkeley; Berkeley CA 94720, USA.; 2 California Institute for Quantitative Biosciences, University of California, Berkeley, CA, 94720, USA; 3 Helen Wills Neuroscience Institute, University of California, Berkeley, Berkeley, CA 94720, USA

## Abstract

Mechanistic target of rapamycin complex 1 (mTORC1) is a nutrient-responsive master regulator of metabolism. Amino acids control the recruitment and activation of mTORC1 at the lysosome via the nucleotide loading state of the heterodimeric Rag GTPases. Under low nutrients, including arginine (Arg), the GTPase activating protein (GAP) complex, GATOR1, promotes GTP hydrolysis on RagA/B, inactivating mTORC1. GATOR1 is regulated by the cage-like GATOR2 complex and cytosolic amino acid sensors. To understand how the Arg-sensor CASTOR1 binds to GATOR2 to disinhibit GATOR1 under low cytosolic Arg, we determined the cryo-EM structure of GATOR2 bound to CASTOR1 in the absence of Arg. Two MIOS WD40 domain β-propellers of the GATOR2 cage engage with both subunits of a single CASTOR1 homodimer. Each propeller binds to a negatively charged MIOS-binding interface on CASTOR1 that is distal to the Arg pocket. The structure shows how Arg-triggered loop ordering in CASTOR1 blocks the MIOS-binding interface, switches off its binding to GATOR2, and so communicates to downstream mTORC1 activation.

## Main Text:

mTORC1 is a master integrator of cell-extrinsic signaling and cell-intrinsic nutrient sensing, and a master regulator of the cellular balance between anabolism and catabolism^1–4^. As such, dysregulation of mTORC1 activity contributes to numerous cancers and metabolic disorders, making mTOR inhibitors a promising therapeutic strategy^5^. The key step in the activation of mTORC1 is its nutrient-regulated recruitment to the lysosomal membrane by the active Rag GTPase-Ragulator complex^6,7^. The Rag-Ragulator complex is composed of RagA or B GTPase, heterodimerized to RagC or D, and tethered to the membrane by the pentameric Ragulator/LAMTOR, whose LAMTOR1 subunit is lipidated^6,8^. In response to nutrients, including Arg, leucine (Leu), glucose and cholesterol, the Rags convert between two stable nucleotide states, inactive (RagA or B^GDP^:RagC or D^GTP^) and active (RagA or B^GTP^:RagC or D^GDP^)^9–12^. The active Rag dimer is responsible for recruiting mTORC1 to lysosomes^13–16^. When cytosolic amino acid levels are low, the Rag-Ragulator complex is inactivated by the GTPase activating protein (GAP) GATOR1, which promotes GTP-to-GDP hydrolysis by RagA/B^13^. The activity of GATOR1 is in turn regulated by the protein complexes GATOR2 and KICSTOR^13,17^. The entire system is targeted to the lysosome principally by the Rag-Ragulator complex^18^. GATOR1, GATOR2, and KICSTOR are not known to directly sense amino acids. Rather, a series of dedicated amino acid sensors that include CASTOR1, Sestrin2 and SAMTOR relay information about amino acids into the pathway by altering the activity of the GATOR1-GATOR2-KICSTOR complexes^19–21^. Understanding how such information is relayed at the structural level is a preeminent question in the regulation of cell metabolism.

GATOR2, a negative regulator of GATOR1, consists of five subunits, WDR59, WDR24, SEH1L, SEC13, and MIOS^13^, that come together to form a higher order cage-like structure that is membrane-less, and shares components and architectural elements with the COP-II cage and the nuclear pore complex^22^. In their apo-states that occur under low amino acids, the Arg sensor CASTOR1 and the Leu sensor Sestrin2 directly bind to GATOR2, preventing the latter from inhibiting the GAP activity of GATOR1^19,20,22^. The CASTOR1 interaction with arginine triggers the dissociation of CASTOR1 from GATOR2, though the structural mechanism for this step is not yet understood^23^. Previous structural studies have uncovered the architecture of GATOR2 and individual nutrient sensors^22–27^. Here, we report the structure of GATOR2 in complex with CASTOR1 in the absence of Arg. By comparing this complex to the pre-existing structures of CASTOR1 in the presence and absence of Arg, we were able to deduce and validate the mechanism whereby Arg binding triggers the release of CASTOR1 from GATOR2 by modulating the conformation of a MIOS-releasing loop and so regulating the accessibilty of the MIOS binding interface of CASTOR1.

### Cryo-EM Structure of the GATOR2-CASTOR1 Complex

**Extended Fig.1:**
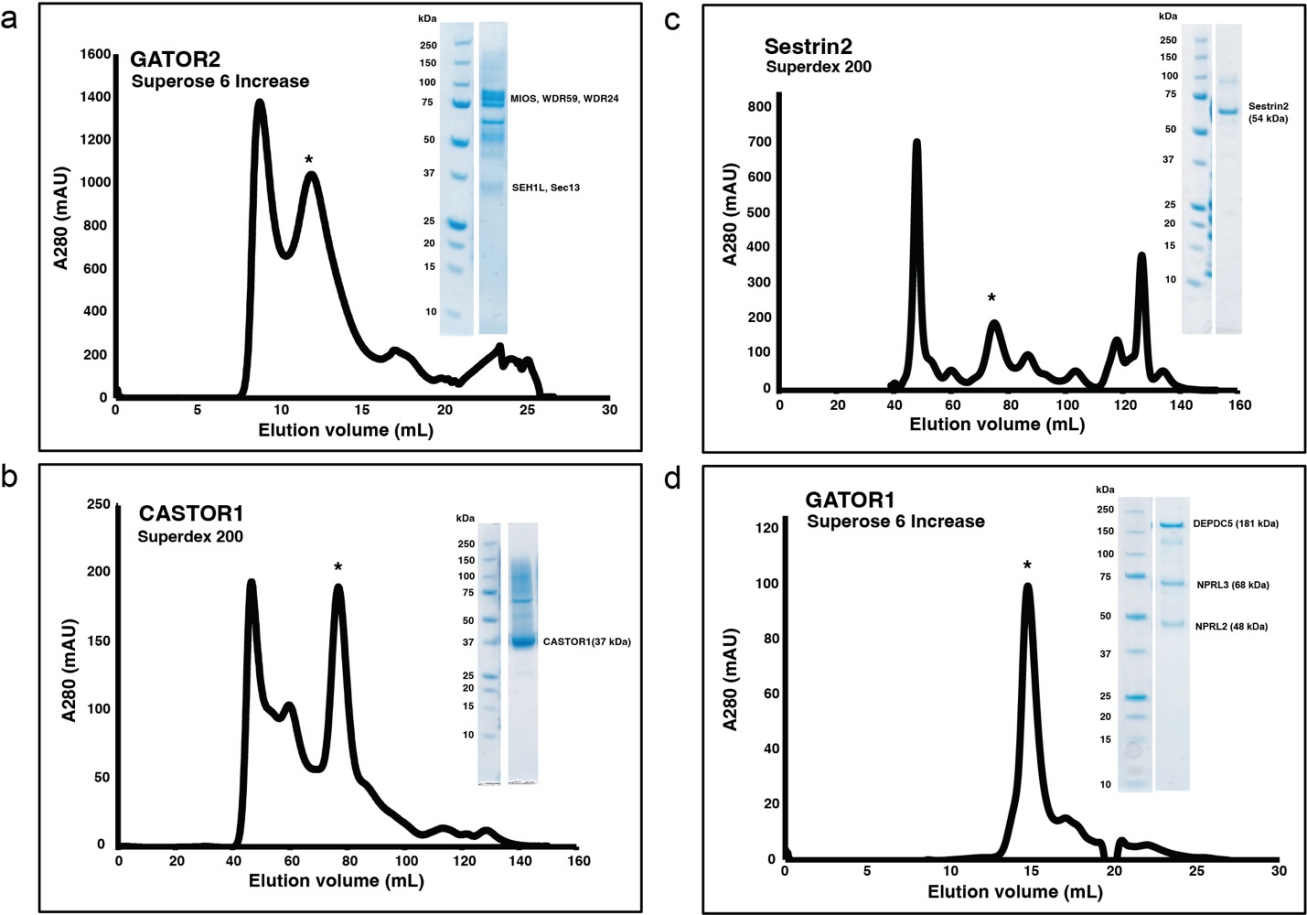
Purification for GATOR2 and CASTOR1. (a) Chromatogram and gel for GATOR2 purification. (b) Chromatogram and gel for CASTOR1 purification. (c) Chromatogram and gel for Sestrin2 purification. (d) Chromatogram and gel for GATOR1 purification.

**Extended Fig.2:**
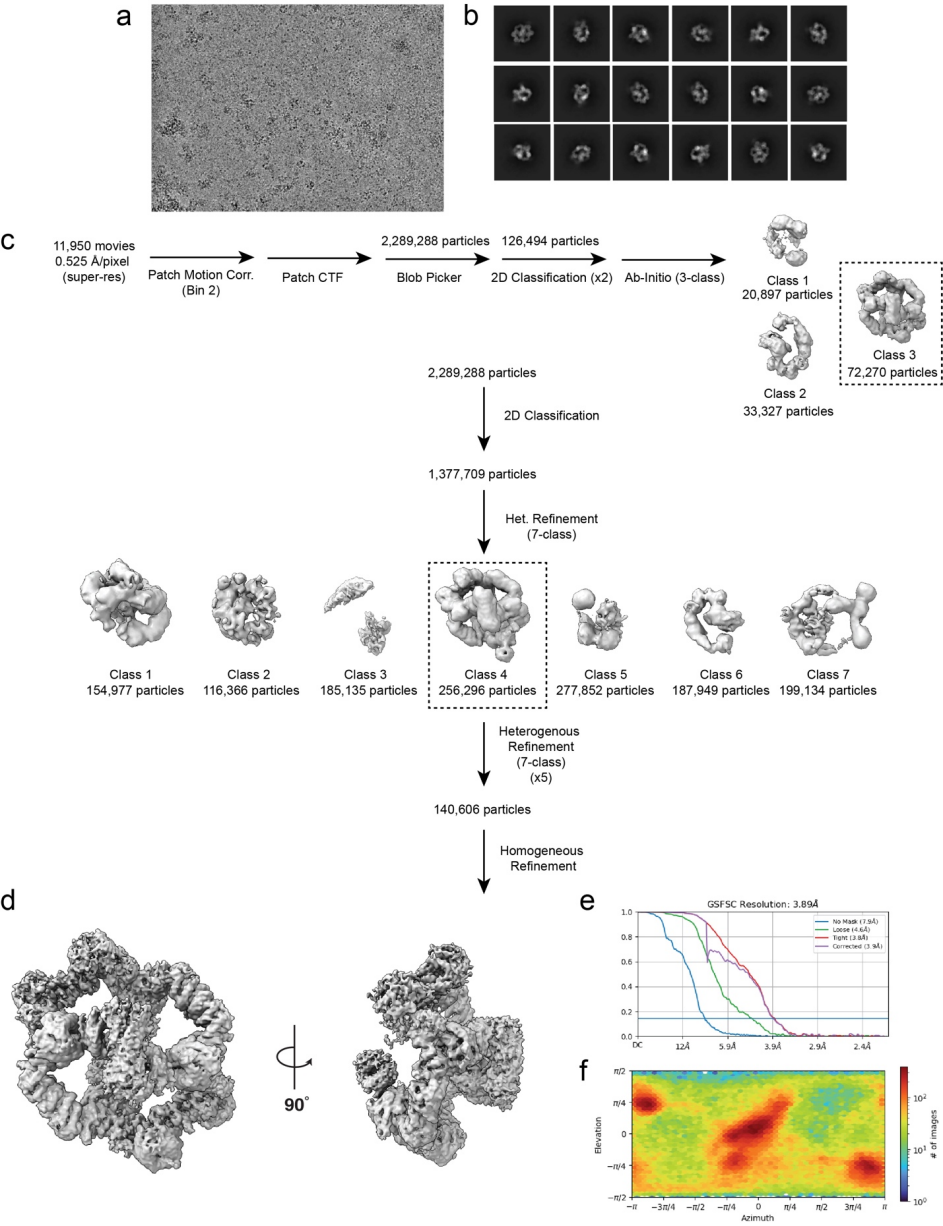
Data Processing Pipeline for GATOR2-CASTOR1 complex. (a) Representative micrograph (b) Representative 2D classes (c) Data processing workflow (d) Overall map for GATOR2-CASTOR1 (e) FSC graph (f) Orientation plot.

**Extended Fig. 3:**
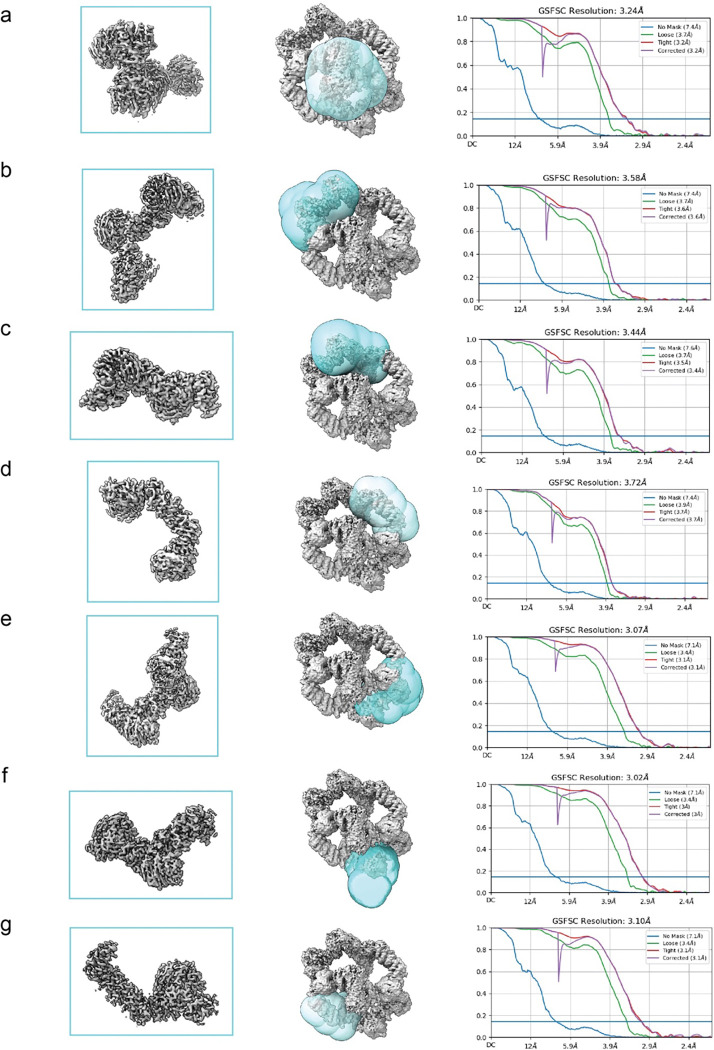
Local Refinement for GATOR2-CASTOR1. (a-g) Local refinement for different sections of complex. Including mask (shown in cyan), FSC graph and resulting map.

**Extended Fig. 4:**
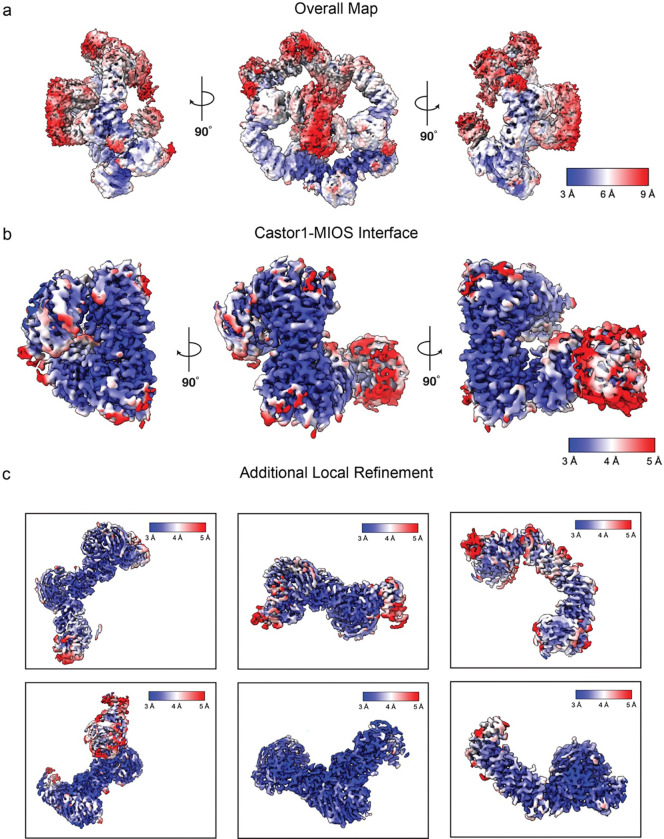
Local resolution estimation. (a) Full complex map (b) CASTOR1-MIOS interface and (c) additional local refinement maps for the complex.

**Extended Fig. 5:**
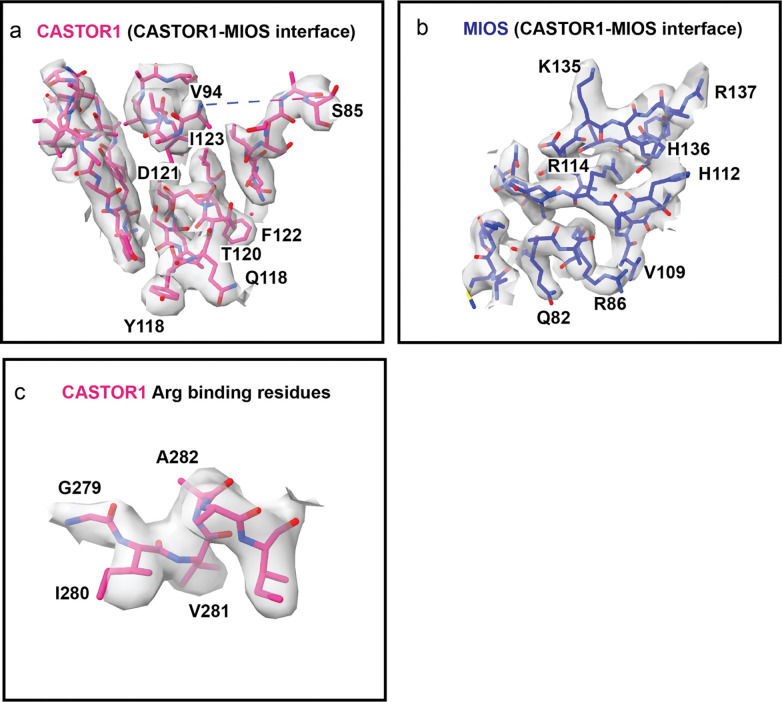
Map to model fit. (a) CASTOR1 at CASTOR1-MIOS interface, (b) MIOS at CASTOR1-MIOS interface (c) CASTOR1 residues near arginine binding pocket.

**Extended Fig. 6:**
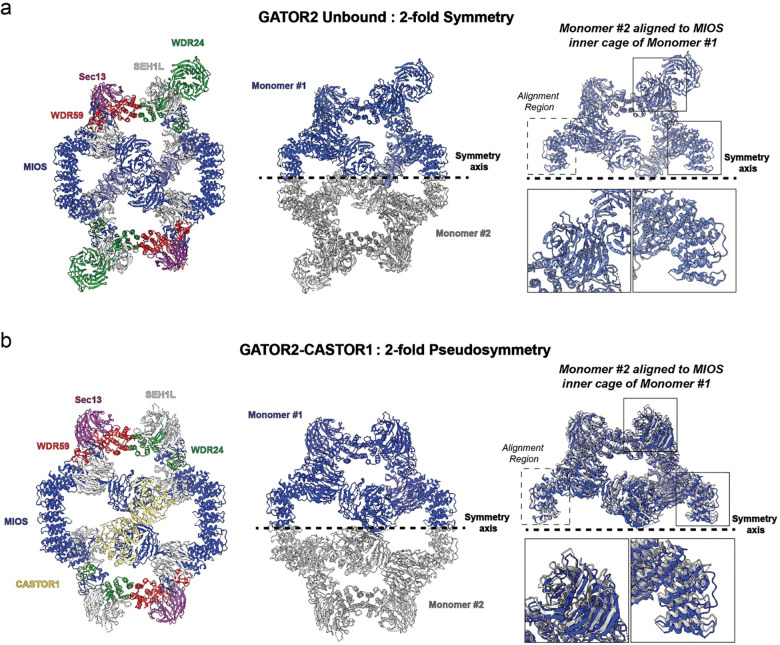
GATOR2 Cage Symmetry. Comparison of cage symmetry for (a) GATOR2 unbound and (b) GATOR2-CASTOR1 complex. For each complex the individual monomers are reflected over the symmetry axis. Regions distal to the alignments region are enlarged for visualization.

**Fig. 1:**
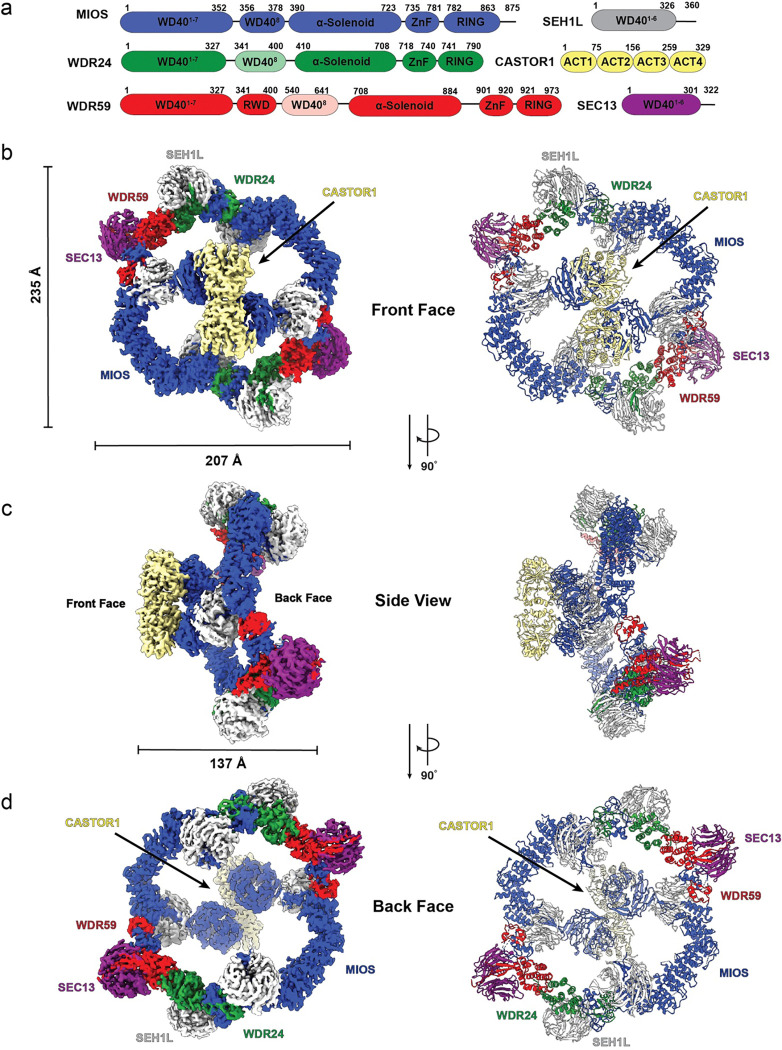
Cryo-EM structure of GATOR2-CASTOR1 complex. (a) Domain organization of subunits within the GATOR2-CASTOR1 structure. Composite map and reconstructed model for the GATOR2-CASTOR1 complex viewing from the (b) front face (c) side view (d) back face. Focused maps for different portions of the complex were combined to generate a composite map containing the highest resolution information for each subunit.

To isolate a stable GATOR2-CASTOR1 complex, we purified wild-type GATOR2 from HEK 293 cells co-transfected with WDR59, WDR24, SEH1L, SEC13, and MIOS. We separately purified a mutant apo-locked CASTOR1^S111A/D304A^ (ref.^23^; hereafter referred to as “CASTOR1^apo^”) from an *E. coli* expression system (Extended Data Fig. 1). The purified GATOR2 and CASTOR1^apo^ were combined, and the cryo-EM structure of GATOR2 bound to CASTOR1^apo^ was determined to an overall resolution of 3.89 Å (Fig. 1 and Extended Data Fig. 2). The resolution of the complex was further improved through local refinement resulting in a resolution range of 3.02 Å - 3.72 Å (Extended Data Fig. 3 and Extended Data Fig. 4). The cryo-EM density was of sufficient quality to generate an atomistic model of the ordered portions of the core cage of GATOR2 and CASTOR1^apo^ (Fig. 1 and Extended Data Fig. 5). The resulting dimensions for the GATOR2-CASTOR1^apo^ complex (hereafter simply “GATOR2-CASTOR1”) were 207 Å × 235 Å × 137 Å. The C2 symmetry of the GATOR2 complex is broken upon CASTOR1 engagement. Analysis of the refined coordinates revealed that the C2 symmetry of the unbound GATOR2 cage is broken in CASTOR1-bound complex (Extended Data Fig. 6), although the two asymmetric units differ by only 1.2 Å r.m.s.d. for Cα atoms.

**Fig. 2:**
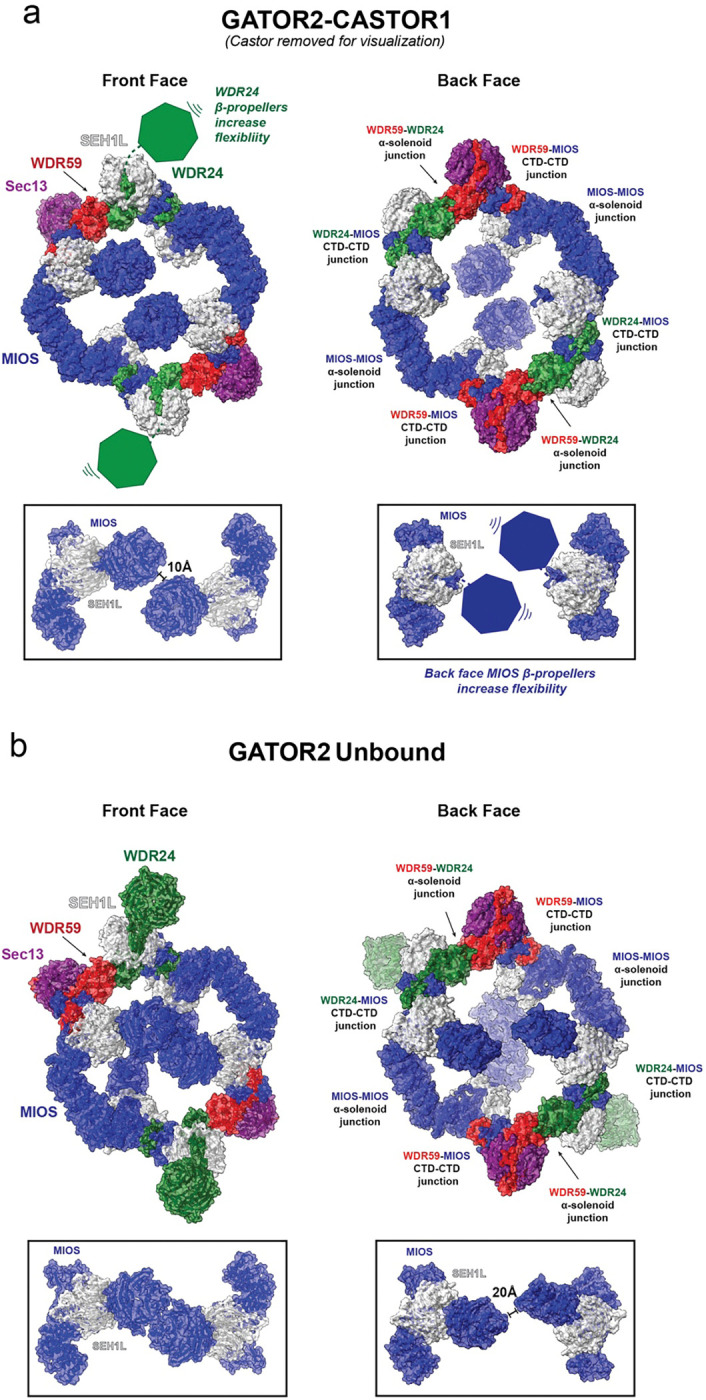
CASTOR1 triggers a structural rearrangement in GATOR2 complex. Comparison of the front and back faces of the (a) GATOR2-CASTOR1 complex and (b) GATOR2^apo^ complex. CASTOR1 is removed for visualization in the GATOR2-CASTOR1 complex. Changes in the MIOS subunits are highlighted in boxes below complex. Key junctions connecting the inner cage are indicated.

As seen in the absence of CASTOR1, GATOR2 assembles into an octagonal scaffold containing four copies of MIOS, two copies of WDR24, two copies of WDR59, two copies of SEC13, and six copies SEH1L^22^. The scaffold is stabilized by four zinc-binding C-terminal domains (CTD) junctions, two of which are formed MIOS-WDR24 and two by MIOS-WDR59, and four junctions formed by interactions between the α-solenoid domains of MIOS-MIOS and WDR24-WDR59^22^ (Fig. 2a). The two MIOS subunits straddling the ‘front’ face of GATOR2 engage the CASTOR1 homodimer (Fig. 1), while the other two back-facing MIOS β-propellers that do not engage CASTOR1 are disordered and are not seen in the final density map and reconstruction (Fig. 1 and 2a).

### CASTOR1 triggers structural rearrangements in GATOR2

The MIOS subunits in GATOR2 play an integral role in the organization of the overall complex core. Comparison with the GATOR2^apo^ unbound structure shows that engagement of CASTOR1 with the front face MIOS β-propellers triggers conformational changes throughout the GATOR2 structure (Fig 2a and 2b, Extended Data Movie 1). Specifically, upon interaction with CASTOR1, the front face MIOS β-propeller pair break their internal interface, pushing apart 10 Å apart and rotating by 16 degrees relative to the MIOS α-solenoid domains of the inner core of the GATOR2 cage (Fig. 2a and 2b) In the GATOR2 unbound conformation, the MIOS interface responsible for interaction with CASTOR1 is exposed and not buried in the β-propeller interface. However, the MIOS β-propellers are too close together to engage both CASTOR1 monomers simultaneously, and thus must reorient in the bound conformation. The back face MIOS β-propellers, in contrast, are already separated by a 20 Å gap in unbound GATOR2 ^22^ (Fig 2b). This gap is too far apart to engage the CASTOR1 dimer and therefore they do not interact with CASTOR1 in the bound complex (Fig. 2b).

**Extended Fig. 7:**
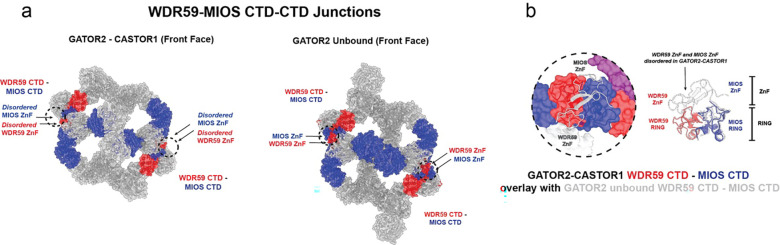
GATOR2 WDR59-MIOS CTD-CTD Junctions. (a) Comparison of the WDR59-MIOS junctions (black dash circle) on GATOR2-CASTOR1 complex and GATOR2 unbound. (b) Close up view of the changes to the WDR59-MIOS CTD junctions. GATOR2 unbound (grey) overlayed with the GATOR2-CASTOR1 (blue and red).

The MIOS subunits are intimately linked with the WDR59 subunits through the MIOS-WDR59 CTD junctions in the GATOR2 complex (Extended Data Fig. 7a). In the GATOR2-CASTOR1 complex, the MIOS β-propeller reorientation pushes the front face SEH1L^MIOS^ subunits outward ~8 Å and shifts the MIOS solenoidal and CTD domains. Compared to GATOR2^apo^, this movement in the front face MIOS solenoidal and CTD domains results in the disordering of residues 757–836 and 890–921 in WDR59 and residues 727–746 and 770–783 in MIOS (Extended Data Fig. 7b). These residues include the zinc finger (ZnF) motif in MIOS and WDR59 (Extended Data Fig. 7b). In the GATOR2^apo^ unbound structure, the ZnF, along with the RING domains in MIOS and WDR59 stabilize the CTD-CTD junctions. Specifically, the MIOS ZnF interacts with Sec13 and the WDR59 ZnF interact with SEH1L^22^. The ZnF contacts are no longer present in the CASTOR1 bound state. However, the RING domains remain intact, preserving the integrity of the GATOR2 cage (Extended Data Fig. 7a).

### The CASTOR1:GATOR2 interface

**Fig. 3:**
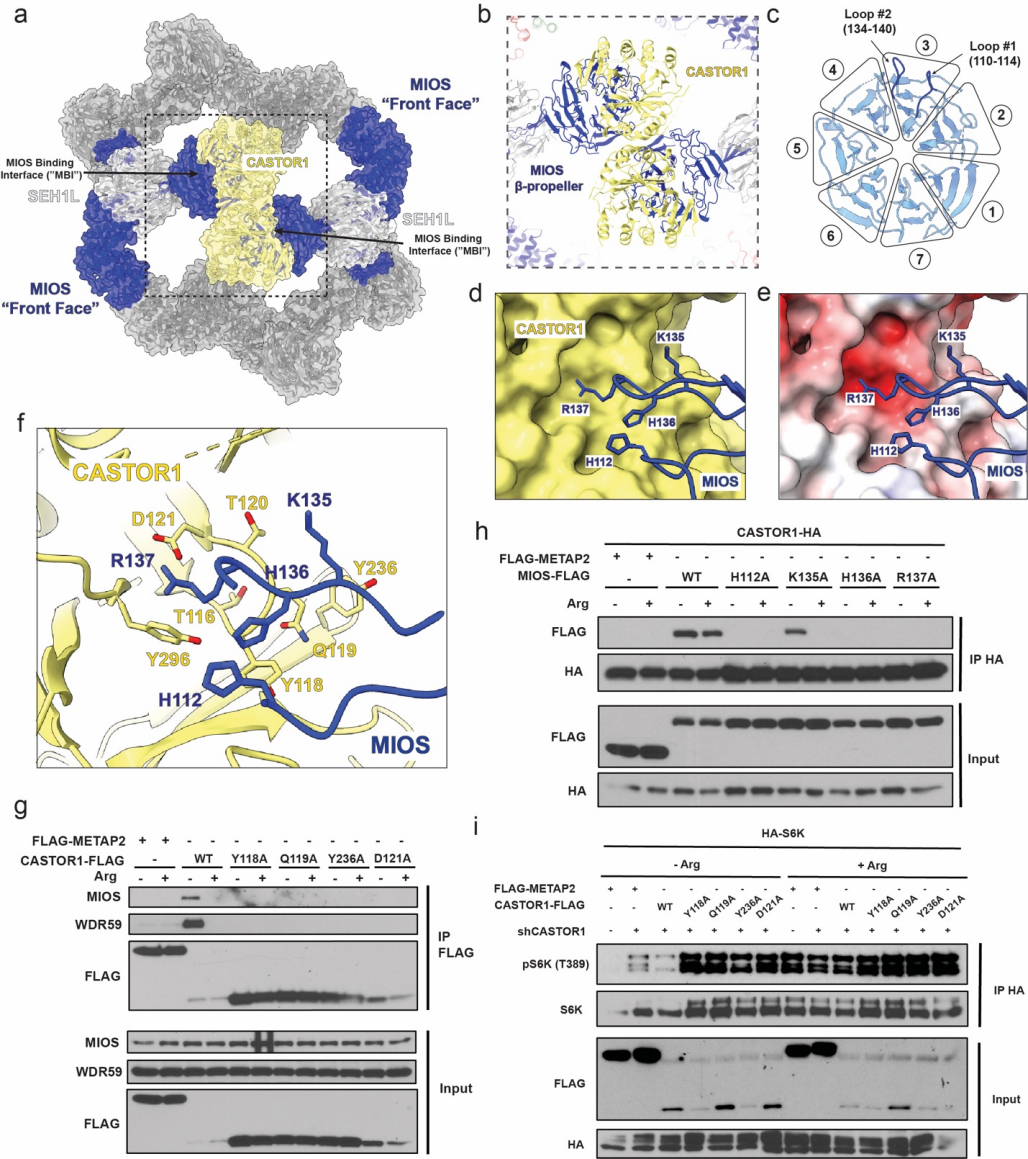
CASTOR1 interacts with MIOS through negatively charged pocket. (a) Overview of GATOR2-CASTOR1 complex. Front face MIOS subunits (blue) interact with CASTOR1 (yellow). (b) Close up view of CASTOR1 interaction with MIOS β-propellers. (c) Blade diagram for a front face MIOS β-propeller highlighting CASTOR1 interacting loops. Close up of the CASTOR1-MIOS interaction shown with (d) CASTOR1 surface view and MIOS ribbon view (e) CASTOR1 surface colored based on electrostatic potential (f) Ribbon view highlighting specific residues in MIOS loops residues 110–114 and 134–140 (blue) interacting with CASTOR1 residues (yellow). (g) HEK-293T cells transiently expressing the indicated FLAG-tagged WT and MIOS binding interface (MBI)-mutant CASTOR1 constructs, or FLAG-tagged METAP2 as a control, were starved of arginine for 50 minutes and, where indicated, restimulated for 10 minutes. FLAG-immunoprecipitates were generated and analyzed by immunoblotting for the indicated proteins. (h) HEK-293T cells transiently expressing CASTOR1-HA and either FLAG-tagged WT MIOS, FLAG-tagged MBI-mutant MIOS constructs or FLAG-tagged METAP2 as a control. Cells were starved of arginine for 50 minutes and, where indicated, restimulated for 10 minutes. HA-immunoprecipitates were generated and analyzed by immunoblotting for the indicated proteins. (i) CASTOR1 knockdown HEK-293T cells transiently expressing the indicated FLAG-tagged WT and MBI-mutant CASTOR1 constructs, or FLAG-tagged METAP2 as a control, were starved of arginine for 50 minutes and, where indicated, restimulated for 10 minutes. Anti-HA-immunoprecipitates were prepared and analyzed by immunoblotting for the indicated proteins and phospho-proteins.

In the GATOR2 bound structure, a single CASTOR1^apo^ homodimer binds across one face of the GATOR2 cage, engaging one MIOS β-propeller domain per CASTOR1^apo^ monomer (Fig. 3a and 3b). The organization of the GATOR2-bound CASTOR1^apo^ dimer is unaltered as compared to the previous CASTOR1^apo^ crystal structure^27^. The CASTOR1^apo^ bound to GATOR2 and the isolated CASTOR1^apo^ crystal structure only differ by 0.7Å rmsd for Cα atoms. Each CASTOR1monomer of consists of four ACT (aspartate kinase, chorismate mutase and TyrA) domains that interact through the interface composed of the ACT1 and ACT4 domains^23,25,26^. The two CASTOR1 monomers in the GATOR2-bound CASTOR1 dimer remain equivalent and the two MIOS binding interfaces as defined by cryo-EM (Fig. 3a) are essentially superimposable on one another. Each interface buries 690 Å^2^ of solvent accessible surface area.

Two MIOS loops are responsible for most of the contacts with CASTOR1^apo^ (Fig. 3b and 3c). Loop #1 (residues 110–114) is located between blade 2 and 3 in the MIOS β-propeller, while loop #2 (residues 134–140) connects two β-sheets in blade 3 (Fig. 3c). The MIOS loop contacts are centered on the basic residues His112, His136, and Arg137, with Lys135 at the edge of the contact region (Fig. 3d–f). These four basic residues engage with a complementary electronegative pocket on the surface of CASTOR1^apo^ centered on Asp121 and also containing Tyr118, Gln119, and Tyr236 (Fig. 3f). To validate the role of the MIOS binding interface in Arg sensing, mutants were generated within the CASTOR1 pocket (D121A, Y118A, Q119A and W236A) and on the two MIOS loops (H112A, K135A, H136A, R137A). The CASTOR1 residues Asp121, Tyr118 and Gln119 and MIOS residue R137 were previously noted to be important for GATOR2 interaction^23,28^. We transiently expressed WT (FLAG-tagged) CASTOR1, or CASTOR1 containing single mutation Y118A, Q119A, D121A or Y236A in HEK-293T cells. As previously reported, in Arg-deprived cells WT CASTOR1-FLAG interacted strongly with endogenous GATOR2 (revealed by immunoblotting for MIOS and WDR59), and this interaction was weakened by Arg refeeding^10^ (Fig. 3g). The interaction between GATOR2 and CASTOR1 was disrupted in cells expressing mutants within the core of the MIOS binding interface on CASTOR1, Y118A and Q119A and D121 as well as cells expressing the mutant Y236A on the periphery of the MIOS binding interface (Fig. 3g). Next, we co-expressed WT MIOS-FLAG or MIOS-FLAG MIOS binding interface mutants (H112A, K135A, H136A and R137A) with WT CASTOR1-HA in HEK-239T cells. Mutations in MIOS loop #1 (H112A) and loop #2 (H136A and R137A) that are central to the MIOS binding interface disrupted GATOR2 interaction with CASTOR1 (Fig. 3h). Mutating a MIOS residue on the periphery of the MIOS binding interface (K135A) has no noticeable effect as compared to WT in Arg-starved conditions. However, the R135A MIOS mutant underwent a more complete dissociation from CASTOR1-HA than the WT protein upon Arg supplementation, suggesting partial destabilization of the interaction with CASTOR1 by this mutation (Fig. 3h). These data validate the functional relevance of both sides of the CASTOR1-MIOS interface.

To understand the role of the CASTOR1-MIOS interaction on downstream mTORC1 activity, we monitored the phosphorylation of the mTORC1 substrate S6K1 in HEK-293T cells depleted for endogenous CASTOR1 via shRNA-mediated knockdown, and reconstituted with transiently expressing CASTOR1, or CASTOR1 containing single mutation Y118A, Q119A, D121A or Y236A. Depletion of endogenous CASTOR1 rendered mTORC1 partially resistant to Arg deprivation, as shown by enhanced phosphorylation of HA-tagged S6K1 in the Arg-depleted sample (Fig. 3i). Co-expressing WT CASTOR1-FLAG restored the suppression of HA-S6K1 phosphorylation by Arg depletion. In contrast to WT, and consistent with their inability to bind to GATOR2, the Y118A, Q119A, D121 and Y236A CASTOR1 mutants failed to restore the normal pattern of HA-S6K1 phosphorylation by Arg (Fig. 3i).

### Mechanism of Arginine-induced CASTOR1 dissociation from GATOR2

**Fig. 4:**
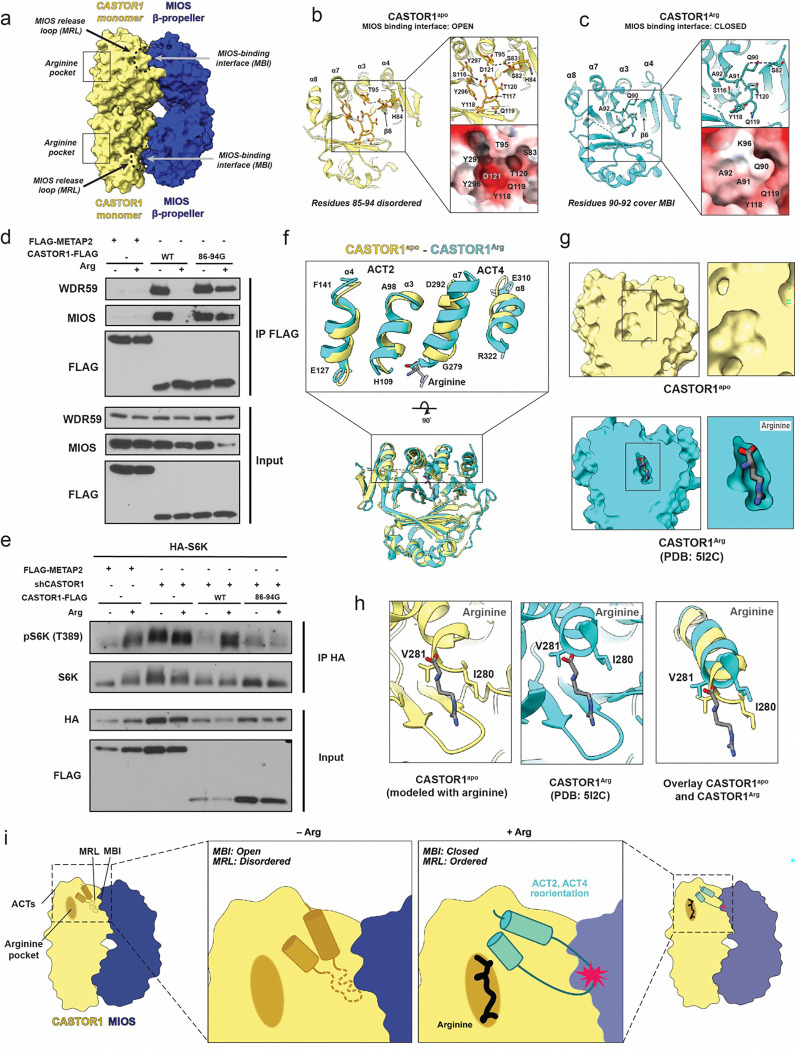
CASTOR1 interaction with arginine triggers closing of GATOR2-interacting pocket. (a) Diagram of CASTOR1 interaction with MIOS β-propellers and location of arginine pocket and MIOS binding interface. (b) Electrostatic surface cartoon of CASTOR1^apo^ and close up of GATOR2-interact pocket. Key residues in CASTOR1 that form pocket are indicated. (c) Electrostatic surface cartoon of CASTOR1^Arg^ and close up of GATOR2-interact pocket. Key residues in CASTOR1 that block pocket are indicated. (d) HEK-293T cells transiently expressing the indicated FLAG-tagged WT and MIOS releasing loop (MRL)-mutant CASTOR1 constructs, or FLAG-tagged METAP2 as a control, were starved of arginine for 50 minutes and, where indicated, restimulated for 10 minutes. FLAG-immunoprecipitates were generated and analyzed by immunoblotting for the indicated proteins. (e) CASTOR1 knockdown HEK-293T cells transiently expressing the indicated FLAG-tagged WT and MRL-mutant CASTOR1 constructs, or FLAG-tagged METAP2 as a control, were starved of arginine for 50 minutes and, where indicated, restimulated for 10 minutes. Anti-HA-immunoprecipitates were prepared and analyzed by immunoblotting for the indicated proteins and phospho-proteins. (f) Overlay of CASTOR1^apo^ (yellow) and CASTOR1^Arg^ (cyan). Rotation in ACT2 and ACT4 α-helices enlarged for visualization. (g) Surface view of CASTOR1^apo^ and CASTOR1^Arg^ arginine binding pocket. CASTOR1^apo^ is modelled with arginine in binding pocket. (h) Ribbon view of arginine binding pocket in CASTOR1^apo^ and CASTOR1^Arg^. (i) Overall model for arginine-dependent CASTOR1 interaction with GATOR2.

The MIOS binding interface on CASTOR1^apo^ is located distal to the Arg binding pocket (Fig. 4a). To understand how information about Arg levels is communicated between the Arg binding pocket and MIOS binding interface, we compared the CASTOR1^apo^ structure obtained through the complex of GATOR2-CASTOR to the crystal structure of CASTOR1 bound to arginine ^23^ (PDB:5I2C) (hereafter referred to as CASTOR1^Arg^) as overlaid on the GATOR2 complex. Examining the electrostatic surface pattern of CASTOR1^apo^ and CASTOR1^Arg^ revealed that only CASTOR1^apo^ has an intact MIOS binding interface for interaction with MIOS (Fig. 4b and 4c). We term the CASTOR1 loop consisting of residues 86–94, which connects β6 and α3 of the ACT2 domain, as the MIOS releasing loop. In CASTOR1^apo^, the MIOS releasing loop is disordered, which exposes the negatively charged MIOS binding interface residues Tyr118, Gln119, Asp121 and Tyr236 (Fig. 4b). In the CASTOR1^Arg^ structure, the residues 90–94 in the MRL are ordered, cover the MIOS binding interface, and sterically block the MIOS-CASTOR1 interaction (Fig. 4c). In essence, the MIOS releasing loop acts as a lid for the MIOS binding interface (Fig. 4a). Disordering of the MIOS releasing loop was previously seen in the isolated CASTOR1^apo^ crystal structure, however, the functional and mechanistic relevance of these residues was not explored^27^.

To test our structural hypothesis that the MIOS releasing loop is responsible for dissociating CASTOR1 from MIOS upon Arg binding, we replaced the MIOS releasing loop with a poly-Gly segment of equal length, CASTOR1^86−94G^, which was designed to be disordered constitutively. The structural hypothesis predicts that the MIOS binding interface of CASTOR1^86−94G^ would remain exposed and functional for MIOS binding even in the presence of Arg. Consistent with the prediction, in HEK293T cells, overexpression of the CASTOR1^86−94G^ mutant constitutively bound to MIOS and suppressed mTORC1 phosphorylation of S6K irrespective of Arg levels (Fig. 4d and 4e).

To explain at the structural level how the negatively charged pocket in CASTOR1^apo^ is linked to binding of arginine in the arginine binding pocket on the other side of CASTOR1, we overlayed the CASTOR1^apo^ and CASTOR1^Arg^ structures. The global architecture of the proteins remained similar, with rotations observed in the α-helices in the ACT2 and ACT4 domains, the portion of CASTOR1 that consists of the arginine binding pocket (Fig. 4f–h). The structural comparison shows how adjustments in the ACT2 and 4 domains can transmit the Arg signal from the Arg pocket on one side of the CASTOR1 monomer to the MIOS releasing loop on the other side of the protein.

### GATOR2 interaction with CASTOR1 and Sestrin2

The leucine sensor, Sestrin2, works in parallel with CASTOR1 to inhibit the activity of GATOR2 and activate GATOR1^9,19^ when cellular amino acid levels are low. The cryo-EM structure of GATOR2-CASTOR revealed the proposed binding sites for Sestrin2 and GATOR1 on WDR24 and WDR59, respectively, remained free. To visualize how CASTOR1 and Sestrin2 simultaneously engages with the GATOR2, we purified WT GATOR2, WT GATOR1, CASTOR1^apo^ and a mutant apo-locked Sestrin2^E451Q/R390A/W444E^ (ref.^23,24^; hereafter referred to as “Sestrin2^apo^”) from an *E. coli* expression system (Extended Data Fig. 1) to generate a cryo-EM sample of GATOR2, GATOR1, Sestrin2 and CASTOR1.

**Extended Fig. 8.**
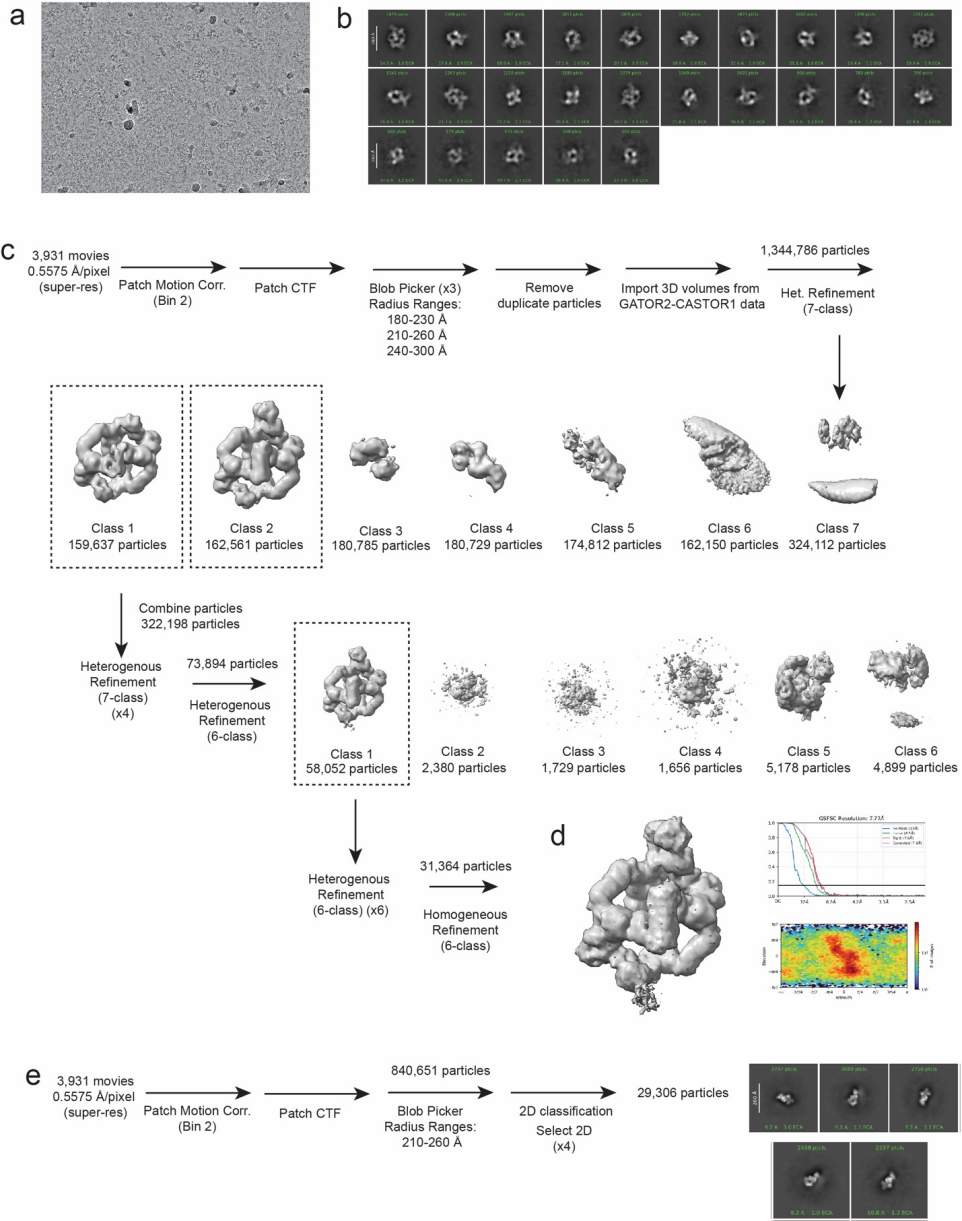
Data Processing Pipeline for GATOR2-CASTOR1-Sestrin2. (a) Representative micrograph (b) Representative 2D classes (c) Data processing workflow (d) Overall map for GATOR2-CASTOR1, FSC graph and orientation plot. (e) Data processing for GATOR1 and representative 2D classes of isolated GATOR1 complex particles.

**Extended Fig. 9:**
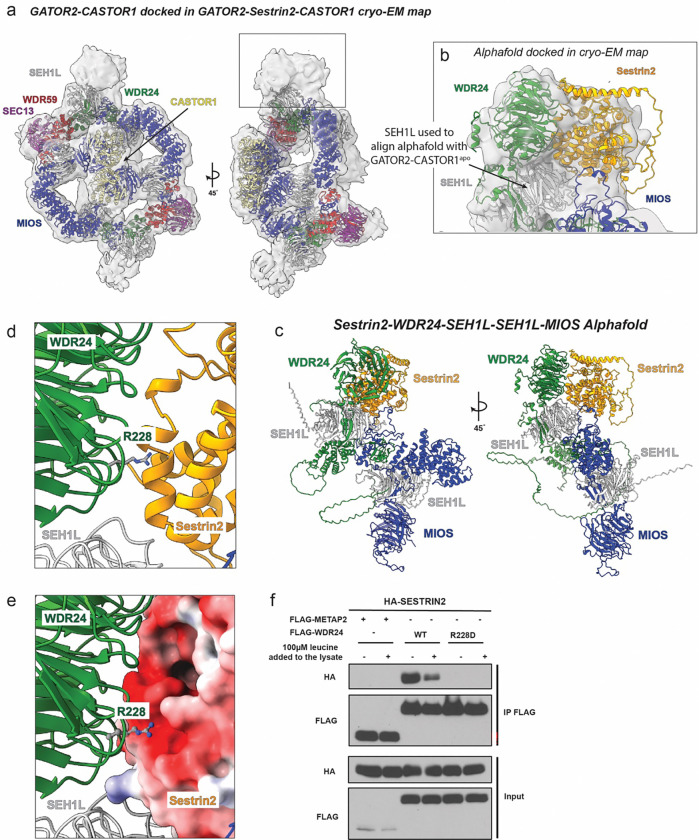
GATOR2-CASTOR1- Sestrin2 interaction. (a) GATOR2-CASTOR1 structure docked into cryo-EM map of GATOR2-CASTOR1-Sestrin2. (b) Close up of GATOR2-CASTOR1-Sestrin2 cryo-EM density fitted with Sestrin2-WDR24-SEH1L-SEH1L-MIOS AlphaFold model (c) Full Sestrin2-WDR24-SEH1L-SEH1L-MIOS AlphaFold model (ipTM = 0.69). Close up of interface between WDR24 (green) and Sestrin2 (orange) in AlphaFold model in (d) ribbon view and (e) surface view colored by electrostatic potential. pLDDT for Arg 228 is 0.89. (f) HEK-293T cells transiently expressing HA-tagged SESTRIN2 along with the indicated FLAG-tagged WDR24 constructs or FLAG-tagged METAP2 as a control were starved of leucine for 50 minutes. Where indicated, leucine was added to the lysates during immunoprecipitation. FLAG-immunoprecipitates were generated and analyzed by immunoblotting for the indicated proteins.

A density map was generated for the complex of GATOR2-CASTOR1-Sestrin2 (Extended Data Fig. 8a–d). GATOR1 present in the sample and was visualized in the data processing but was not detected bound to the GATOR2-CASTOR1-Sestrin2 complex (Extended Data Fig. 8e). GATOR2-CASTOR1^apo^ docked into the final map suggesting Sestrin2 was compatible with the GATOR2- CASTOR1^apo^ cage alterations (Extended Data Fig 9a). Unassigned density was visible in the map at the location of the WDR24 β-propeller and adjacent to it (Extended Data Fig 9a). We generated an AlphaFold model of Sestrin2 with a portion of one GATOR2 asymmetric unit containing one copy of WDR24, two copies of SEH1L and one copy of MIOS (Extended Data Fig. 9c). The AlphaFold model was fitted in the cryo-EM map through alignment with the SEH1L subunit connected to WDR24 in GATOR2-CASTOR1 (Extended Data Fig. 9b). In the AlphaFold model, Sestrin2 makes significant contact with the WDR24 β-propeller, as suggested by previous studies^24,28^, burying 1,195 Å^2^ of surface area. The interface in the AlphaFold model was analyzed and WDR24 Arg228 made critical contacts with the negatively charged Sestrin2 surface (Extended Data Fig. 9d and 9e). To validate this interaction, a mutant was generated in the WDR24 β-propeller (R228D). We transiently expressed WT HA-tagged Sesrin2 along with WT FLAG-tagged WDR24, or WDR24 containing single mutation R228D in HEK-293T cells.

As previously reported, in Leu-deprived cells WT HA-Sestrin2 interacted strongly with the GATOR2 subunit WDR24, and this interaction was weakened by Leu refeeding^10^ (Extended Data Fig. 9f). The interaction between GATOR2 and Sestrin2 was disrupted in cells expressing WDR24 R228D suggesting the relevance of the interface observed in the AlphaFold model (Extended Data Fig. 9f). Our cryo-EM and AlphaFold model revealed the location of Sestrin2 binding that is compatible with CASTOR1 interaction with GATOR2. While we only observed one stably bound copy of Sestrin2 via cryo-EM, it remains possible that an additional copy of Sestrin2 could interact with the cage given the second copy of WDR24. Together, these data show that Sestrin2 and CASTOR1 bind to the same conformational state of GATOR2, consistent with a common mechanism for downstream regulation of GATOR1.

## Discussion

The new structure presented here is consistent with a model that links CASTOR1 interaction with Arg to changes in the GATOR2-CASTOR1 interaction, and reveals a mechanism for Arg-induced dissociation of CASTOR1 from GATOR2 leading to mTORC1 activation. Here, we directly visualized the CASTOR1 MIOS-binding interface. Previous structural comparison of isolated apo- and arginine-bound CASTOR1 crystal structures noted two missing loop regions in the apo-CASTOR1 structure ^27^. The functional implications of this change had been unclear, but can now be understood in light of the structure of the GATOR2-CASTOR1 complex. The Arg binding pocket and the MIOS binding interface reside on opposite faces of CASTOR1, and are connected by the α-helices of the ACT2 and ACT4 domains. In low Arg conditions, the GATOR2 pocket is exposed while the arginine binding pocket is covered. Upon increases in Arg levels, Arg enters the binding pocket and signals through conformational changes in the α-helices to the opposite face of CASTOR1. This leads to ordering of the MIOS releasing loop, occlusion of the MIOS binding interface, and so to the release of CASTOR1 from GATOR2 (Fig. 4i).

We found that one CASTOR1 dimer engages two MIOS WD40 domains on the front face of GATOR2 even though two other MIOS subunits are present on the back face of the cage. The inability of CASTOR1 to bind to the back face MIOS dimer is explained by the greater separation of these domains. At 20 Å apart in the unbound GATOR2 structure, it may be sterically impossible to draw the back face MIOS β-propeller pair together to the 10 Å separation needed to bind the CASTOR1 dimer. This prevents the formation of a 2:4 GATOR2 asymmetric unit:CASTOR1 monomer complex. Thus, while the overall cage remains intact, conformational changes extend over the entire cage and break exact C2 symmetry.

The critical remaining question is how the Arg signal is transduced to GATOR1. In yeast, the counterparts of GATOR1 and 2 (the SEA complex) interact directly. The cryo-EM structure of the SEA has been determined^29^, yet the precise mechanism of GATOR1 GAP regulation is still unclear, even in yeast. A third protein complex, KICSTOR, is present in mammals that does not exist in yeast^17^. KICSTOR has been shown to engage both GATOR1 and GATOR2 and regulate their activity^17,30^. The structure of the GATOR2-CASTOR1-Sestrin2 triple complex determined here shows that these factors can bind simultaneously, a result consistent with the expectation that, physiologically, low-nutrient states should involve simultaneous depletion of multiple amino acid species. Now that the key question as to how amino acid binding regulates sensor engagement has been answered, at least for CASTOR1 and Arg, the central question going forward is how GATOR1 GAP activity is regulated by the combined action of GATOR2-CASTOR1-Sestrin2 and KICSTOR. GATOR2 interactions with Sestrin2, CASTOR1, and GATOR1 are not mutually exclusive, and the findings here thus set the stage to ultimately answer this question.

How the Rag GTPases interconvert between the active and inactive nucleotide states^9–12^ is at the very heart of understanding nutrient regulation of mTORC1. The nucleotide state of RagC/D is important primarily for regulation of non-canonical mTORC1 substrates, of which the MiT-TFE transcription factors are the best characterized ^31^. The structural pathway for regulation of the RagC/D nucleotide state by the FLCN-FNIP GAP complex has been worked out in large part^32–35^. In contrast, despite its critical importance for both canonical and non-canonical mTORC1 signaling, regulation of the nucleotide state of RagA/B remains incompletely understood. Structural analysis of the GATOR1 GAP complex^36,37^ and GATOR2^22^ is making strides towards a full structural and mechanistic explanation of this central event. The work presented here adds another important piece to the puzzle, bringing us that much closer to a complete structural view of how the RagA/B branch of mTORC1 signaling is regulated.

## Methods

### Cloning and Protein Purification:

#### GATOR2 purification

Codon optimized DNA encoding all five subunits of GATOR2 (MIOS, WDR59, WDR24, SEH1L and Sec13) was synthesized by Twist Biosciences and subcloned into the pCAG vector. The construct with MIOS was engineering to include a N-terminal tandem-STREP-FLAG tag. HEK293-GNTI cells were co-transfected with 1mg DNA with equal amount of all five GATOR2 subunits and 4 mg P.E.I per 1L of cells at 2E6 cells/ml. Cells were harvested after 48 hours and pelleted at 2000 ×g for 20 min at 4 °C.

Cell pellets were resuspended in 30 mL lysis buffer (25 mM HEPES pH 7.5, 500 mM NaCl, 2 mM MgCl_2_, 10% glycerol, 1 mM TCEP, 1 protease inhibitor tablet (Roche) per 50 mL, 1 mM PMSF) and dounce homogenized prior to 1 hour incubation with 1% DDM:CHS (1:10) at 4 °C. The lysate was centrifuged at 37,000 ×g for 35 minutes at 4 °C. The supernatant was incubated with ~3–4 mL of Strep-Tactin Sepharose resin for 12–15 hours rocking at 4 °C. The resin was washed with 20 mL high salt wash buffer A (25 mM HEPES, 500 mM NaCl, 2 mM MgCl_2_, 1 mM TCEP, 50 mM Arginine, 50 mM Glutamic Acid, 1 mM ATP, pH 7.4, 0.03% DDM/CHS), 20 mL low salt wash buffer B (25 mM HEPES, 200 mM NaCl, 2 mM MgCl_2_, 1 mM TCEP, 50 mM Arginine, 50 mM Glutamic Acid, 1 mM ATP, pH 7.4, 0.03% DDM/CHS), 20 mL low salt (no ATP) wash buffer C (25 mM HEPES, 200 mM NaCl, 2 mM MgCl_2_, 1 mM TCEP, 50 mM Arginine, 50 mM Glutamic Acid, pH 7.4, 0.03% DDM/CHS), and 20 mL low salt (no ATP, no DDM:CHS) wash buffer D (25 mM HEPES, 200 mM NaCl, 2 mM MgCl_2_, 1 mM TCEP, 50 mM Arginine, 50 mM Glutamic Acid, pH 7.4). GATOR2 was eluted from the Strep-Tactin Sepharose resin using 20 mL elution buffer (25 mM HEPES, 200 mM NaCl, 2 mM MgCl_2_, 1 mM TCEP, 50 mM Arginine, 50 mM Glutamic acid, pH 7.4, 4mM desthiobiotin). Eluted protein was concentrated to 1 mL using a Milipore Amicon Ultra Centrifugal Filter and subjected to gel filtration using a Superose 6 Increase 10/300 and buffer containing 25 mM HEPES, 200 mM NaCl, 2 mM MgCl_2_, 1 mM TCEP.

#### CASTOR1^apo^ purification

Codon optimized DNA encoding CASTOR1 S111A/D304A was synthesized by Twist Biosciences and subcloned into the pET-28a+ vector containing an N-terminal 6X-His tag. The vector containing 6X-His-CASTOR1^apo^ was transformed into BL21(DE3) cells. Cells were grown at 37 °C until the optical density (OD) reached 0.6. Protein production was induced using 0.2 mM IPTG at 18 °C for 14–16 hours. Cells were pelleted via centrifugation at 3500 ×g for 20 minutes.

Cell pellets were resuspended in ~50 mL lysis buffer (30 mM Tris-HCL, 200 mM NaCl, 1mM TCEP, 1 mM PMSF) and lysed via sonication for 5 minutes, 2 seconds ON, 2 seconds OFF. The lysate was centrifuged at 37,000 ×g for 35 minutes at 4 °C. The supernatant was incubated with ~3 mL HisPur Ni-NTA resin (Thermo Scientific) for 1–2 hr rocking at 4 °C. The resin was washed with ~150 mL wash buffer (30 mM Tris-HCL, 200 mM NaCl, 30 mM imidazole, 1mM TCEP) before elution with ~80 mL elution buffer (30 mM Tris-HCL, 200 mM NaCl, 200 mM imidazole, 1mM TCEP). The protein was concentrated using Milipore Amicon Ultra Centrifugation Filter to 1 mL. The concentrated protein was subjected to gel filtration using HiLoad 16/600 Superdex 200 pg column and buffer containing (10mM HEPES, pH 7.5, 100mM NaCl, 0.5mM TCEP).

#### Sestrin2^apo^ purification

Codon optimized DNA encoding Sestrin2 E451Q/ R390A/ W444E was synthesized by Twist Biosciences and subcloned into the pET-28a+ vector containing an N-terminal 6X-His tag. The vector containing 6X-His-Sestrin2^apo^ was transformed into BL21(DE3) cells. Cells were grown at 37 °C until the optical density (OD) reached 0.7. Protein production was induced using 0.2 mM IPTG at 18 °C for 14–16 hours. Cells were pelleted via centrifugation at 3500 ×g for 20 minutes.

Cell pellets were resuspended in ~50 mL lysis buffer (50mM Potassium Phosphate pH 8.0, 500mM NaCl, 30mM imidazole, 3mM BME, and 1mM PMSF) and lysed via sonication for 5 minutes, 2 seconds ON, 2 seconds OFF. The lysate was centrifuged at 37,000 ×g for 35 minutes at 4 °C. The supernatant was passed through ~5 mL HisPur Ni-NTA resin (Thermo Scientific), collected and passed through 2x more. The resin was washed with ~150 mL wash buffer (50mM Potassium Phosphate pH 8.0, 500mM NaCl, 30mM imidazole, 3mM BME, and 1mM PMSF) before elution with ~50 mL elution buffer (50mM Potassium Phosphate pH 8.0, 150mM NaCl, 250mM imidazole, 3mM BME). The protein was dialyzed using SnakeSkin Dialysis Tubing (10K MWCO) (Thermo Scientific) in 4L of buffer containing 10mM potassium phosphate and 100mM NaCl at 4 °C for 14–16 hours. The protein was passed through 5mL HiTrap SP HP cation exchange column (Cytiva) and the flow through was collected and saved. The protein was concentrated using Milipore Amicon Ultra Centrifugation Filter to 1 mL. The concentrated protein was subjected to gel filtration using HiLoad 16/600 Superdex 200 pg column and buffer containing (10mM Tris-HCl pH 8.0, 150mM NaCl, 0.1mM EDTA and 0.5mM TCEP).

#### GATOR1 purification

HEK293-GNTI cells were co-transfected with 1mg DNA encoding the GATOR1 subunits GST-tagged DEPDC5, NPRL2 and NPRL2 at a 1:2:2 ratio and 4 mg P.E.I per 1L of cells at 2E6 cells/ml. Cells were harvested after 48 hours and pelleted at 2000 ×g for 20 min at 4 °C. Cell pellets were resuspended in 30 mL lysis buffer (25 mM HEPES pH 7.5, 130 mM NaCl, 2.5 mM MgCl_2_, 2mM EGTA, 1% Triton-X 0.5 mM TCEP, and 1 protease inhibitor tablet (Roche) per 50 mL) and incubated for 1 hour at 4 °C. The lysate was centrifuged at 37,000 ×g for 35 minutes at 4 °C. The supernatant was incubated with ~3–4 mL of Glutathione Sepharose resin for 3 hours rocking at 4 °C. The resin was washed with 15 mL lysis buffer, 15 mL high salt lysis buffer (25 mM HEPES pH 7.5, 500 mM NaCl, 2.5 mM MgCl_2_, 2mM EGTA, 1% Triton-X 0.5 mM TCEP), 10mL lysis buffer and 15mL gel filtration buffer (25mM HEPES pH 7.5, 130mM NaCl, 2.5mM MgCl_2_, 0.5mM TCEP). The column was sealed and an additional 5mL of gel filtration and TEV protease was added. The column was incubated with TEV protease overnight for cleavage. The protein was eluted from the column with 15 mL gel filtration buffer and concentrated to 1 mL using a Milipore Amicon Ultra Centrifugal Filter. The sample was subjected to gel filtration using a Superose 6 Increase 10/300 for a final polishing step with buffer containing 25 mM HEPES pH 7.5, 130 mM NaCl, 2 mM MgCl_2_, 0.5 mM TCEP).

### Cryo-EM Grid Preparation and Imaging:

#### GATOR2-CASTOR1^apo^

Purified GATOR2 was concentrated to 0.45 mg/mL. 3-fold molar excess of CASTOR1 was added and incubated for 45 minutes on ice and immediately froze on grids. 3 μl sample was deposited onto freshly glow-discharged (PELCO easiGlow, 30 s in air at 15 mA and 0.4 mbar) holey carbon grids (C-flat: 2/1–3Cu-T). FEI Vitrobot Mark IV was used to blot grids for 3 seconds with a blot force of 20 (Whatman 597 filter paper) at 4°C and 100 % humidity and subsequentially plunged into liquid ethane. The Titan Krios G3i microscope equipped with a Gatan Quantum energy filter (slit width 20 eV) and a K3 summit camera at a defocus of −1.0 to −2.0 μm was used to record 11,950 movies. Automated image acquisition was performed using SerialEM ^1^ recording four movies per 2 μm hole with image shift. Image parameters are summarized in Extended Table 1.

#### GATOR2-CASTOR1^apo^- Sestrin2^apo^ – GATOR1

Purified GATOR2 was concentrated to 0.45 mg/mL. 3-fold molar excess of CASTOR1, 2-fold molar excess of Sestrin2, and 3-fold molar excess of GATOR1 was added and incubated for 45 minutes on ice and immediately froze on grids. 3 μl sample was deposited onto freshly glow-discharged (PELCO easiGlow, 30 s in air at 15 mA and 0.4 mbar) holey carbon grids (C-flat: 2/1–3Cu-T). FEI Vitrobot Mark IV was used to blot grids for 3 seconds with a blot force of 20 (Whatman 597 filter paper) at 4°C and 100 % humidity and subsequentially plunged into liquid ethane. The Talos Arctica microscope equipped with a Gatan K3 camera at a defocus of −1.0 to −2.0 μm was used to record 3,931 movies. Automated image acquisition was performed using SerialEM ^1^ recording 2 movies per 2 μm hole with image shift. Image parameters are summarized in Extended Table 1.

### Cryo-EM Data Processing:

The data processing workflow for GATOR2-CASTOR1^apo^ is summarized in Extended Data Fig.1. In short, raw movies were imported into cryosparc2 v4.3.1 ^2^. Patch Motion Corr. was used for motion correction and Patch CTF estimated (multi) was used for CTF determination. Cryosparc blob picker with a diameter range of 200Å-280Å was used to generate 3,467,659 which was inspected to trim the particle set to 2,289,288 particles. Particles were extracted with a box size of 560×560 pixels in cryosparc2. A series of 2D classifications followed by an ab-initio-reconstruction was used to generate three reference maps. The resulting 3D maps were used in addition to maps generated from prior datasets to resort all 2,289,288 particles after a round of 2D classification to remove obvious ‘junk’. The final particle set contained 140,606 particles and a round of homogenous refinement resulted in a 3.89Å map at 0.143 FSC. Masks were generated surrounding various subunits within the complex using UCSF ChimeraX and imported into cryosparc2 v3.3.1 where it was lowpass filtered and dilated^3^ (Extended Data Fig.2). The masks were used for subsequent local refinement and resulted in improvements of the map between 3.02 Å – 3.72 Å (Extended Data Fig.2 and Extended Data Fig.3).

The data processing workflow for GATOR2-CASTOR1^apo^- Sestrin2^apo^-GATOR1 is summarized in Extended Data Fig.9. In short, raw movies were imported into cryosparc2 v4.3.1 ^2^. Patch Motion Corr. was used for motion correction and Patch CTF estimated (multi) was used for CTF determination. Cryosparc blob picker with a diameter range of 180Å-230Å, 210Å-260Å and 240Å-300Å were used to generate 1,344,786. Particles were extracted with a box size of 560×560 pixels in cryosparc2. Volumes from GATOR2-CASTOR1^apo^ corresponding to full complex and junk classes were imported and used for subsequent rounds of heterogenous refinement. The final particle set contained 31,364 particles and a round of homogenous refinement resulted in a 7.77Å map at 0.143 FSC. The final map revealed density for Sestrin2 bound to the GATOR2-CASTOR1^apo^ cage but not GATOR1. 2D classification was used to visualize the quality of the final particle set. Additionally, the particles picked using the 210Å-260Å were sorted in 2D for GATOR1 particles. 2D classes corresponding to GATOR1 were visualized but not bound to the GATOR2 complex.

### Atomic Model Building and Refinement:

A composite map for GATOR2-CASTOR1 was generated in UCSF ChimeraX^3^ by aligning the local refinement maps to the overall map and combining the best portions of the maps. The coordinates for GATOR2 (7UHY) and arginine bound CASTOR1 (5I2C) were rigid body fitted into the composite map in UCSF ChimeraX^3^. To account for movement of the GATOR2 subunits, the structure was separated into its individual subunits and each subunit was rigid body fitted independently into the map. The MIOS subunit undergoes the largest conformational change upon CASTOR1 binding. Due to this, the MIOS subunits of GATOR2 were broken down into three smaller portions encompassing the residues 43–380, 387–728 and 783–863. Each of these smaller portions were rigid body fit into the map. The rigid body fit subunits were combined into a new model for further refinement. The model was refined using iterative rounds of Phenix real-space refinement^4–6^. In between rounds of refinement, the model was manually inspected for fit in the composite map. Residues outside of the map region were manually removed using COOT. The CASTOR1 mutations (S111A and D304A) were manually incorporated following the first iteration of refinement using COOT.

### Arginine Binding Pocket Analysis:

Analysis of the CASTOR1 arginine binding pockets was performed using the CASTp program^7^.

### Structure prediction using AlphaFold3:

#### GATOR2-CASTOR1-Sestrin2 prediction

The structure model of Sestrin2, WDR24, MIOS, and 2 copies of SEH1L was generated using AlphaFold3^8^. The confidence of the predicted models were assessed by pLDDT. The Sestrin2-WDR24-MIOS-SEH1L-SEH1L was overlayed with each WDR24 subunit of the GATOR2-CASTOR1 cryo-EM structure to generate a GATOR2-CASTOR1-Sestrin2 full complex prediction.

### Antibodies and chemicals

Reagents were obtained from the following sources: antibodies to MIOS (13557S), WDR59 (53385S), FLAG (14793S), HA (3724S), S6K1 (2708S), phospho-T389-S6K1 (9234S) from Cell Signaling Technology.

FLAG-M2 affinity gel (A2220) and individual powders of amino acids from Sigma Aldrich; Pierce anti-HA magnetic beads (88836), Pierce protease inhibitor tablets, EDTA-free (A32965) and hygromycin B (10687010) from Thermo Fisher Scientific; RPMI 1640 without glucose and amino acids (R9010–01) from US Biologicals.

### Mammalian Cell Culture

Adherent HEK293T human embryonic kidney cells were cultured in DMEM base media supplemented with 10% (v/v) heat-inactivated fetal bovine serum, penicillin (100U/mL) and streptomycin (100μg/mL). Cells were maintained in a humid atmosphere at 37°C and 5% CO_2_. Cells were routinely tested for mycoplasma contamination using MycoAlert Mycoplasma Detection kit (Lonza, LT07–318).

### Lentivirus production and infection

Lentiviruses were prepared by co-transfecting pLKO.1 constructs along with psPAX2 and pMD2G packaging plasmids into HEK293T cells using the PEI transfection method. Viral supernatant was collected 48h post-transfection and filtered using 0.45μm PES syringe filter. The virus was then concentrated using Lenti-X concentrator (Takara Bio, 631232) according to manufacturer’s protocol, and stored at −80°C.

Short-hairpin oligonucleotides (shRNAs) directed against CASTOR1 (TRCN0000284010), MIOS (TRCN0000303645) or Luciferase (TRCN0000072243, used as a non-targeting control) were cloned into the pLKO.1 lentiviral vector (The RNAi Consortium, Broad Institute) according to the manufacturer’s instructions.

For lentivirus infection, HEK293T cells were seeded along with concentrated virus and 8μg/ml polybrene (Millipore, TR-1003-G). After 24h, the media was changed to fresh media supplemented with hygromycin B for selection. Experiments were performed 7 days post-infection.

### Transfections, amino acid starvation, cell lysis, immunoprecipitation and western blot

#### Castor1 interaction with GATOR2

Transient transfection of cDNAs into HEK293T cells was performed using the calcium phosphate transfection method. Briefly, 2.10^6^ HEK293T cells were plated in 10cm dishes. 24h after, cells were transfected with the appropriate pRK5-based cDNA in the following amounts: 2000ng METAP2, 3000ng FLAG-MIOS, 2000ng CASTOR1-FLAG, 2ng HA-S6K. The total amount of plasmid DNA was normalized to 5000ng with empty pRK5 for each transfection. 6h after, media containing the transfection mix was replaced with fresh media. Experiments were performed 36h after.

For arginine starvation or restimulation, cells were incubated with arginine free RPMI for 50min and, when indicated, restimulated with 1.15mM arginine for 10min. After the indicated treatments, cells were rinsed once with ice-cold PBS and lysed in lysis buffer (10mM sodium-pyrophosphate, 10mM sodium-beta-glycerophosphate, 40mM HEPES, 4mM EDTA, 1% Triton X-100, pH 7.4, supplemented with one EDTA-free protease inhibitor tablet per 50 ml). After 30min at 4°C under gentle agitation, cell lysates were cleared by centrifugation at 17,000 × g for 10min, 4°C. Protein concentrations were normalized across samples by BCA assay. Equal amounts of proteins were incubated with 30μL of pre-washed anti-HA magnetic beads or FLAG-M2 affinity gel for 2h at 4°C with end-over-end rotation. The immunoprecipitates were washed three times with lysis buffer before denaturation by addition of 50μL sample buffer and incubation at room temperature for 16h, 65°C for 10min or 95°C for 5min. Samples were resolved by 4–20% SDS–PAGE and analyzed by immunoblotting.

#### Sestrin2 interaction with GATOR2

Transient transfection of cDNAs into HEK293T cells was performed using the calcium phosphate transfection method. Briefly, 2.10^6^ HEK293T cells were plated in 10cm dishes. 24h after, cells were transfected with the appropriate pRK5-based cDNA in the following amounts: 1000ng METAP2, 3000ng FLAG-MIOS, 4000ng FLAG-WDR24, 500ng HA-SESTRIN2, 2000ng CASTOR1-FLAG, 2ng HA-S6K. The total amount of plasmid DNA was normalized to 5000ng with empty pRK5 for each transfection. 6h after, media containing the transfection mix was replaced with fresh media. Experiments were performed 36h after. For arginine or leucine starvation, cells were incubated with arginine or leucine free RPMI for 50min. For restimulation, arginine (1.15mM) was added to the media for 10min before lysis. Leucine (0.38mM) was added to the lysates for 2h during immunoprecipitation.

### cDNA cloning

Codon optimized and shRNA-resistant gene fragments (Twist Biosciences) for CASTOR1-FLAG and FLAG-MIOS were cloned into the pRK5 vector. CASTOR1 and MIOS mutants were generated using the site-directed mutagenesis QuikChange method. Briefly, two overlapping primers containing the desired mutation in the center were designed. After PCR amplification, products were DpnI digested and transformed into chemically competent *E.coli*. Mutations were confirmed by Sanger sequencing (Quintara Biosciences).

### qPCR confirmation shCASTOR1

RNA was extracted from HEK293T cells using the Aurum Total RNA Mini kit (BIORAD, Cat#732–6820). Equal amounts of RNA were reverse-transcribed using the iScript Reverse Transcription Supermix kit (BIORAD, Cat#177–8840). The resulting cDNA was amplified by qPCR using the SsoAdvanced Universal SYBR Gren Supermix (BIORAD, Cat#172–5270). Data were analyzed using the 2-ΔΔCt methods and normalized by the housekeeping genes ACTB and HPRT1

The following primers were used: ACTB forward, 5’-GGACTTCGAGCAAGAGATGG-3’; ACTB reverse 5’-AGCACTGTGTTGGCGTACAG-3’; HPRT1 forward, 5’-TGACACTGGCAAAACAATGCA-3’; HPRT1 reverse 5’-GGTCCTTTTCACCAG CAAGCT-3’; CASTOR1 forward, 5’-GCCACCACCCTCATAGATGT-3’; CASTOR1 reverse 5’-AGGAGGTCACTGGGGAACTT-3’.

## Extended Data

**Extended Fig. 10:**
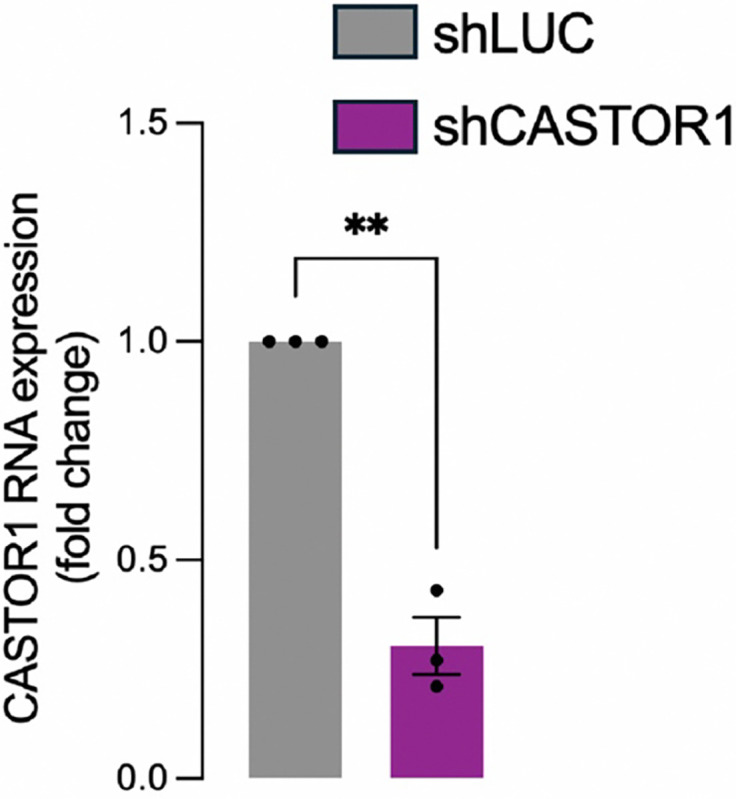
qPCR confirmation shCASTOR1. qPCR against CASTOR1 performed in HEK293T transduced with a shRNA targeting Luciferase (shLUC) or a shRNA targeting CASTOR1. Data were normalized using ACTB and HPRT1 as housekeeping genes.

**Extended Data Table 1:** Cryo-EM data acquisition and image processing.

	GATOR2-CASTOR1 complex	GATOR2-CASTOR1-Sestrin2 Complex

**Data acquisition**		
Microscope	Titan Krios	Talos Arctica
Voltage (kV)	300	200
Camera	GATAN K3	GATAN K3
Magnification	165,000	36,000
Pixel size (Å)	0.525 (super-resolution)	0.558 (super-resolution)
Cumulative exposure (e^−^/Å^2^)	50	50
Energy filter slit width (eV)	20 eV	
Defocus range (μm)	−1.0 to −2.0	−1.0 to −2.0
Automation software	SerialEM	SerialEM
Exposure navigation	Image Shift	Image Shift
Number of movies	11,950	3,931
**Image processing**		
Initial picked particles (no.)	2,289,288	1,344,786
Final refined particles (no.)	140,606	31.364
Map resolution (Å) FSC threshold	Overall: 3.02-3.72, Interface: 3.24 0.143	Overall: 7.77

**Extended Data Table 2:** GATOR2-CASTOR1 coordinate model refinement and assembly

PDB access code EMDB	
**Refinement**	
Software	Phenix 1.19
Refinement target (Å)	3.24 (interface) 3.89 (overall)
Non-hydrogen atoms	43,315
Residues	6,081
GATOR2 reference PDB	7UHY
CASTOR1 reference PDB	5I2C
**Map-model statistics**	
R.M.S deviations	
Bond lengths (Å)	0.002
Bonds angles (Å)	0.453
**Validation**	
Molprobitity	1.56
Clash score	8
Rotamer ouliers (%)	0.03
Cβ outliers (%)	0.00
CaBLAM outliers (%)	1.23
Ramachandran	
Favored (%)	0.03
Allowed (%)	2.59
Outlier (%)	97.37
**Final model composition**	
Number of chains	18
Number of Residues	6,081
B-factors	
Protein (min/max/average)	23.7/140.37/74.06
